# Variation in the Phosphoinositide 3-Kinase Gamma Gene Affects Plasma HDL-Cholesterol without Modification of Metabolic or Inflammatory Markers

**DOI:** 10.1371/journal.pone.0144494

**Published:** 2015-12-10

**Authors:** Martin Kächele, Anita M. Hennige, Jürgen Machann, Anja Hieronimus, Apostolia Lamprinou, Fausto Machicao, Fritz Schick, Andreas Fritsche, Norbert Stefan, Bernd Nürnberg, Hans-Ulrich Häring, Harald Staiger

**Affiliations:** 1 Department of Internal Medicine, Division of Endocrinology, Diabetology, Angiology, Nephrology and Clinical Chemistry, University Hospital Tübingen, Tübingen, Germany; 2 Institute for Diabetes Research and Metabolic Diseases of the Helmholtz Centre Munich at the University of Tübingen, Tübingen, Germany; 3 German Centre for Diabetes Research (DZD), Tübingen, Germany; 4 Department of Diagnostic and Interventional Radiology, Section on Experimental Radiology, University Hospital Tübingen, Tübingen, Germany; 5 Department of Internal Medicine, Division of Nutritional and Preventive Medicine, University Hospital Tübingen, Tübingen, Germany; 6 Department of Experimental and Clinical Pharmacology and Toxicology, Division of Pharmacology and Experimental Therapy, University Hospital Tübingen, Tübingen, Germany; Northeast Ohio Medical University, UNITED STATES

## Abstract

**Objective:**

Phosphoinositide 3-kinase γ (PI3Kγ) is a G-protein-coupled receptor-activated lipid kinase mainly expressed in leukocytes and cells of the cardiovascular system. PI3Kγ plays an important signaling role in inflammatory processes. Since subclinical inflammation is a hallmark of atherosclerosis, obesity-related insulin resistance, and pancreatic β-cell failure, we asked whether common genetic variation in the PI3Kγ gene (*PIK3CG*) contributes to body fat content/distribution, serum adipokine/cytokine concentrations, alterations in plasma lipid profiles, insulin sensitivity, insulin release, and glucose homeostasis.

**Study Design:**

Using a tagging single nucleotide polymorphism (SNP) approach, we analyzed genotype-phenotype associations in 2,068 German subjects genotyped for 10 *PIK3CG* SNPs and characterized by oral glucose tolerance tests. In subgroups, data from hyperinsulinaemic-euglycaemic clamps, magnetic resonance spectroscopy of the liver, whole-body magnetic resonance imaging, and intravenous glucose tolerance tests were available, and peripheral blood mononuclear cells (PBMCs) were used for gene expression analysis.

**Results:**

After appropriate adjustment, none of the *PIK3CG* tagging SNPs was significantly associated with body fat content/distribution, adipokine/cytokine concentrations, insulin sensitivity, insulin secretion, or blood glucose concentrations (p>0.0127, all; Bonferroni-corrected α-level: 0.0051). However, six non-linked SNPs displayed at least nominal associations with plasma HDL-cholesterol concentrations, two of them (rs4288294 and rs116697954) reaching the level of study-wide significance (p = 0.0003 and p = 0.0004, respectively). More precisely, rs4288294 and rs116697954 influenced HDL2-, but not HDL3-, cholesterol. With respect to the SNPs’ in vivo functionality, rs4288294 was significantly associated with *PIK3CG* mRNA expression in PBMCs.

**Conclusions:**

We could demonstrate that common genetic variation in the *PIK3CG* locus, possibly via altered *PIK3CG* gene expression, determines plasma HDL-cholesterol concentrations. Since HDL2-, but not HDL3-, cholesterol is influenced by *PIK3CG* variants, PI3Kγ may play a role in HDL clearance rather than in HDL biogenesis. Even though the molecular pathways connecting PI3Kγ and HDL metabolism remain to be further elucidated, this finding could add a novel aspect to the pathophysiological role of PI3Kγ in atherogenesis.

## Introduction

Phosphoinositide 3-kinases (PI3Ks) are a family of enzymes which catalyze the phosphorylation of intracellular phosphoinositides, an important step in many signaling pathways mediating cell growth, proliferation, and differentiation [1]. PI3Ks are divided into three different classes based on their structure, function, and substrate specificity [2]. Class-I kinases are the best characterized PI3Ks catalyzing the phosphorylation of phosphatidylinositol 4,5-bisphosphate to phosphatidylinositol 3,4,5-trisphosphate. All class-I PI3Ks are heterodimers consisting of two subunits: one for the catalytic function, the other one acting as an adapter or regulatory protein. Class-I catalytic subunits have a molecular mass of about 110 kDa (referred to as p110 subunits). Different genes encode for the four different p110 subunits, i.e., *PIK3CA*, *PIK3CB*, *PIK3CG*, and *PIK3CD*, that give rise to the four class-I PI3K isoforms termed PI3Kα, -β, -γ, and -δ. With respect to their function, class-I PI3Ks are subdivided into class-I_A_ (PI3Kα, -β, and -δ) and class-I_B_ (PI3Kγ) kinases [3]. Class-I_A_ kinases are associated with a regulatory subunit termed p85 and are activated by receptor tyrosine kinases and the GTPase Ras (e.g., PI3Kα) [4] or by G-protein-coupled receptors via the Gβγ subunit of heterotrimeric G-proteins and Rho-family GTPases (e.g., PI3Kβ) [5,6]. Prominent signaling pathways including class-I_A_ kinases are the insulin and insulin-like growth factor signaling pathways mediating activation of Akt/protein kinase B and other downstream mediators which are important for the regulation of glucose metabolism and cell proliferation.

PI3Kγ, as the only class-I_B_ kinase, heterodimerizes with a regulatory p87 or p101 subunit and is activated by G-protein-coupled receptors via Gβγ and Ras [7–9]. PI3Kγ is mainly expressed in leukocytes and cells of the cardiovascular system and plays an important signaling role in inflammatory responses. Chronic low-grade, so-called subclinical, inflammation in turn is a major driver of metabolic diseases and their comorbidities, such as cardiovascular disease, fatty liver disease, and type-2 diabetes [10–12]. Notably, blockade of PI3Kγ either by genetic means (knockout) or by pharmacological inhibition revealed beneficial effects on disease outcome in mouse models of inflammatory disorders like diet-induced obesity, hepatic steatosis, lupus erythematodes, and atherosclerosis [13–17].

Human *in vivo* studies addressing the role of PI3Kγ in inflammation and inflammation-related metabolic diseases are still lacking. Therefore, we asked whether common genetic variation (minor allele frequency [MAF] ≥0.05) in the PI3Kγ gene *PIK3CG* exists and whether it affects body fat content and/or distribution, serum cytokine and adipokine concentrations, plasma lipid profiles, insulin sensitivity, insulin release, and glucose homeostasis. To this end, we applied a tagging single nucleotide polymorphism (SNP) approach in a total of 2,068 metabolically characterised subjects at increased risk for type-2 diabetes from the Tübingen Family (TÜF) study for type-2 diabetes.

## Material and Methods

### Study participants

The TÜF study currently comprises more than 2,500 non-related German Caucasians at increased risk for type-2 diabetes, i.e., non-diabetic subjects with a family history of type-2 diabetes, a body mass index (BMI) ≥27 kg/m^2^, impaired fasting glycaemia, and/or previous gestational diabetes [18]. All participants underwent physical examination, routine blood tests, and oral glucose tolerance tests (OGTTs). Furthermore, we assessed the medical history, smoking status, and alcohol consumption habits. From the TÜF study, 2,068 subjects with complete anthropometric data sets and documented absence of medication known to influence glucose tolerance, insulin sensitivity, or insulin secretion were genotyped. In the overall study population, 2,066 complete OGTT data sets, 1,243 adiponectin and leptin measurements, and 383 interleukin 6 (IL-6), tumour necrosis factor α (TNF-α), and monocyte chemoattractant protein 1 (MCP-1) measurements were available. Furthermore, data from hyperinsulinaemic-euglycaemic clamps (HECs), magnetic resonance spectroscopy (MRS) of the liver, whole-body magnetic resonance imaging (MRI), and intravenous glucose tolerance tests (IVGTTs) derived from partially overlapping subgroups of 499, 481, 361, and 306 individuals, respectively, were analysed. The clinical characteristics of the overall study population and the major subgroups are given in Table 1. In a very small subgroup of the overall study population (N = 34), high-density lipoprotein (HDL)-cholesterol fractionation data were available from an earlier investigation [19]. This subgroup was not analysed in the earlier investigation [19]. From 29 randomly selected participants, peripheral blood mononuclear cells (PBMCs) were prepared und subjected to gene expression analysis.

**Table 1:** Clinical data of the overall study population and the major subgroups.

Parameter	Overall (N = 2,068)	Adipokines (N = 1,243)	Cytokines (N = 383)	HEC (N = 499)	MRS (N = 481)	MRI (N = 361)	IVGTT (N = 306)
N (women/men)	1,334/734	814/429	248/135	268/231	306/175	222/139	175/131
Age (y)	39.6 ±12.6	39.0 ±12.3	40.4 ±12.6	39.8 ±12.0	44.2 12.0	45.4 ±11.6	44.6 ±11.3
BMI (kg/m²)	30.8 ±9.5	29.4 ±8.5	28.4 ±6.0	27.2 ±5.5	30.3 ±5.0	29.9 ±5.3	29.2 ±5.3
Body fat content (%)	33.5 ±12.8	31.7 ±11.6	30.9 ±9.5	28.3 ±9.6	34.6 ±9.6	33.0 ±8.9	31.8 ±8.8
NGT/IFG/IGT/IFG+IGT	1,443/258/200/167	890/135/118/100	271/37/44/31	382/39/46/32	310/68/55/48	230/43/48/40	204/31/43/28
Glucose, fasting (mmol/L)	5.16 ±0.54	5.13 ±0.54	5.10 ±0.52	5.02 ±0.54	5.27 ±0.49	5.24 ±0.50	5.17 ±0.49
2-h Glucose (mmol/L)	6.36 ±1.61	6.31 ±1.63	6.44 ±1.70	6.18 ±1.70	6.82 ±1.52	6.90 ±1.58	6.79 ±1.66
Leptin (ng/mL)	-	28.0 ±31.5	-	-	-	-	-
Adiponectin (μg/mL)	-	14.4 ±7.4	-	-	-	-	-
IL-6 (pg/mL)	-	-	0.882 ±0.840	-	-	-	-
TNF-α (pg/mL)	-	-	2.75 ±5.41	-	-	-	-
MCP-1 (pg/mL)	-	-	181.7 ±91.6	-	-	-	-
ISI, HEC (*10^6^ L*kg^-1^*min^-1^)	-	-	-	0.084 ±0.052	-	-	-
IHL (% signal)	-	-	-	-	1.27 ±1.09	-	-
TAT (% body weight)	-	-	-	-	-	30.5 ±9.2	-
VAT (% body weight)	-	-	-	-	-	3.33 ±1.71	-
AIR (pmol/L)	-	-	-	-	-	-	934 ±617

Data are given as counts or means ±SD. AIR–acute insulin response; AUC–area under the curve; BMI–body mass index; C-Pep–C-peptide; CRP–C-reactive protein; Glc–glucose; HEC–hyperinsulinaemic-euglycaemic clamp; IFG–impaired fasting glycaemia; IGT–impaired glucose tolerance; IHL–intrahepatic lipids; IL-6—interleukin-6; Ins—insulin; ISI–insulin sensitivity index; IVGTT–intravenous glucose tolerance test; MCP-1 –monocyte chemoattractant protein 1; MRI–magnetic resonance imaging; MRS–magnetic resonance spectroscopy; NGT–normal glucose tolerance; OGTT–oral glucose tolerance test; TAT–total adipose tissue; TNF-α –tumor necrosis factor α; VAT–visceral adipose tissue

### Ethics statement

All participants gave informed written consent to the study which adhered to the Declaration of Helsinki. The study protocol was approved by the Ethics Committee of the Medical Faculty of the Eberhard Karls University Tübingen.

### Determination of body fat content/distribution

Waist circumference (in cm) as a crude proxy of abdominal fat mass was measured at the midpoint between the lateral iliac crest and the lowest rib in the supine position. BMI as a crude measure of whole-body adiposity was calculated as weight divided by squared height (in kg/m^2^). The percentage of body fat was measured by bioelectrical impedance (BIA-101, RJL systems, Detroit, MI, USA). To precisely quantify total adipose tissue (TAT) and visceral adipose tissue (VAT) contents, whole-body MRI was performed [20]. The intrahepatic lipid (IHL) content was quantified by localized stimulated echo acquisition mode ^1^H-MRS [21].

### OGTT

After an overnight fasting period of 10 h, a standardised 75-g OGTT was performed. For the determination of plasma glucose, serum insulin and C-peptide levels, venous blood samples were drawn at baseline and at time-points 30, 60, 90, and 120 min of the OGTT.

### IVGTT and HEC

In study participants who agreed to undergo both the IVGTT and the HEC, the IVGTT was performed after a 10-h overnight fast and prior to the HEC according to the Botnia regimen [22]. After having collected baseline samples at time-points -10 and -5 min, a glucose bolus of 0.3 g/kg body weight was given intravenously at time-point 0. Blood samples for the measurement of plasma glucose and serum insulin were obtained at time-points 2, 4, 6, 8, 10, 20, 30, 40, 50, and 60 min of the IVGTT. The HEC was started after a 10-h overnight fast or, if combined with an IVGTT, at time-point 60 min after the IVGTT glucose bolus by giving a primed infusion of short-acting human insulin (40 mU/m^2^/min) for 120 min. Concomitantly, variable infusion of glucose (20%) was started to clamp the plasma glucose concentration at fasting levels. Blood samples for the measurement of plasma glucose were obtained at 5-min intervals. Serum insulin levels were measured at baseline (or prior to the glucose bolus of the IVGTT) and at the steady state (the last 30 min) of the clamp.

### Laboratory measurements

Plasma glucose levels (in mmol/L) were measured with a bedside glucose analyser (glucose oxidase method, Yellow Springs Instruments, Yellow Springs, OH, USA). Serum insulin and C-peptide concentrations (in pmol/L, both) were determined by commercial chemiluminescence assays for ADVIA Centaur (Siemens Medical Solutions Diagnostics, Fernwald, Germany). Plasma triglycerides, total cholesterol, HDL and low-density lipoprotein (LDL) cholesterol, and wide-range C-reactive protein (in mg/dL, all) were measured using the ADVIA 1800 clinical chemical analyser. HDL2- and HDL3-cholesterol concentrations (in mg/dL) were determined after ultracentrifugation, as described earlier [23]. Apolipoprotein A1 (apoA1; in mg/dL) was measured by immunonephelometry. Blood cell counts were realized on the ADVIA 2120 haematology analyser (Siemens Healthcare Diagnostics, Eschborn, Germany). Serum adiponectin (in μg/mL) and leptin concentrations (in ng/mL) as well as TNF-α, IL-6, and MCP-1 concentrations (in pg/mL, all) were determined by enzyme-linked immunosorbent assays (adiponectin and leptin–Linco Research, St. Charles, MO, USA; TNF-α and IL-6 –R&D Systems, Wiesbaden-Nordenstadt, Germany; MCP-1 –Bender MedSystems, Vienna, Austria). Plasma free fatty acid (FFA) levels (in μmol/L) were determined enzymatically (NEFAC kit, WAKO Chemicals, Neuss, Germany).

### Calculations

Insulin sensitivity was calculated from fasting data, the OGTT, and the HEC. Based on fasting data, homoeostasis model assessment of insulin resistance (HOMA-IR) was calculated as c(Glc_0_[mmol/L])*c(Ins_0_[mU/L])/22.5 with c = concentration, Glc = glucose, and Ins = insulin [24]. The OGTT-derived insulin sensitivity index (ISI-OGTT) was calculated as 10,000/{c(Glc_0_[mmol/L])*c(Ins_0_[pmol/L])*c(Glc_mean_[mmol/L])*c(Ins_mean_[pmol/L])}^½^ [25]. The HEC-derived ISI (ISI-HEC) was calculated as glucose infusion rate necessary to maintain euglycaemia during the last 20 min (steady state) of the clamp (in μmol/kg/min) divided by the steady-state insulin concentration (in pmol/L). Insulin secretion was calculated from the OGTT and the IVGTT. From the OGTT, one insulin-based and one C-peptide-based index were calculated as area under the curve (AUC)_0-30 min_ Insulin/AUC_0-30 min_ Glucose and AUC_0-120 min_ C-Peptide/AUC_0-120 min_ Glucose, respectively [26]. AUC_0-30 min_ Insulin/AUC_0-30 min_ Glucose was calculated as {c(Ins_0_[pmol/L])+c(Ins_30_[pmol/L])}/{c(Glc_0_[mmol/L])+c(Glc_30_[mmol/L])}. AUC_0-120 min_ C-Peptide/AUC_0-120 min_ Glucose was calculated according to the trapezoid method as ½{½c(C-Pep_0_[pmol/L])+c(C-Pep_30_[pmol/L])+c(C-Pep_60_[pmol/L])+c(C-Pep_90_[pmol/L])+½c(C-Pep_120_[pmol/L])}/½{½c(Glc_0_[mmol/L])+c(Glc_30_[mmol/L])+c(Glc_60_[mmol/L])+c(Glc_90_[mmol/L])+½c(Glc_120_[mmol/L])} with C-Pep = C-peptide. From the IVGTT, insulin secretion was calculated as acute insulin response (AIR) according to the trapezoid method as ½{½c(Ins_0_[pmol/L])+c(Ins_2_[pmol/L])+c(Ins_4_[pmol/L])+c(Ins_6_[pmol/L])+c(Ins_8_[pmol/L])+½c(Ins_10_[pmol/L])}. The gender-dependent Framingham risk score for cardiovascular disease risk was calculated and converted to 10-year risk categories according to the instructions of the National Institutes of Health (www.nhlbi.nih.gov/health-pro/guidelines/current/cholesterol-guidelines/quick-desk-reference-html/10-year-risk-framingham-table).

### Selection of tagging SNPs

To identify tagging SNPs, a genomic area of 43.8 kb on human chromosome 7q22 including the complete *PIK3CG* gene (11 exons, 10 introns) and 2 kb of its 5’-flanking (promoter) region was analysed *in silico*. Based on genomic data from the Central European (CEU) population of the 1000 Genomes Project (http://browser.1000genomes.org/index.html), we identified 10 representative SNPs that tag all the other common SNPs (MAF ≥0.05) in this region with an r^2^ ≥0.8 (100% coverage) using the tagger analysis tool of Haploview (http://www.broadinstitute.org/scientific-community/science/programs/medical-and-population-genetics/haploview/haploview). These tagging SNPs were rs4727666 (A/G) in the 5’-flanking region, rs3823963 (T/A) in intron 1, rs1129293 (C/T) in exon 3 (Ser675Ser), rs17401277 (C/T) in intron 6, rs59813697 (A/C), rs4288294 (C/T), rs849405 (A/G), rs116697954 (C/T), and rs2037718 (C/G) in intron 10, and rs10216210 (G/C) in exon 11 (3’-untranslated region). Importantly, the *PIK3CG* gene is flanked 68 kb upstream by a long intergenic non-coding RNA gene (*RNA5SP236*) and 137 kb downstream by the *PRKAR2B* gene, and no obvious linkage blocks were observed that span the *PIK3CG* locus and one of these adjacent genes.

### Genotyping

DNA was isolated from whole blood using a commercial kit (NucleoSpin, Macherey & Nagel, Düren, Germany). Genotyping of the tagging SNPs was performed by mass spectrometry using the massARRAY genotyping platform (Sequenom, Hamburg, Germany) and the manufacturer’s iPLEX software. SNPs rs849405, rs2037718, and rs10216210 resisted massARRAY multiplex assay design and were, therefore, genotyped by allelic discrimination using commercial TaqMan assays (Applied Biosystems, Foster City, CA, USA). The call rates were ≥96%. The genotyping results were validated in 50 randomly selected subjects by bidirectional sequencing, and no deviations were observed.

### PBMC isolation and quantitative real-time polymerase chain reaction (qPCR)

PBMCs were isolated from whole blood by density gradient centrifugation using the Ficoll-based Lymphocyte Separation Medium 1077 from PAA Laboratories (Cölbe, Germany). Cells were washed with phosphate-buffered saline, lysed with RLT buffer (Qiagen, Hilden, Germany), and homogenized using QIAshredder (Qiagen). Total-RNA was isolated with RNeasy columns (Qiagen), treated with RNase-free DNase I, and reverse transcribed into cDNA using the Transcriptor First Strand cDNA Synthesis kit from Roche Diagnostics (Mannheim, Germany). QPCR of *PIK3CG*, *CETP* (encoding cholesteryl ester transfer protein), *PLTP* (encoding phospholipid transfer protein), *LCAT* (encoding lecithin-cholesterol acyltransferase), *SCARB1* (encoding scavenger receptor B1), and *RPS13* (encoding ribosomal protein S13) mRNA expression was performed in technical duplicates on a LightCycler 480 (Roche Diagnostics) using Probes Master and fluorescent reporter probes from the Universal Probe Library (Roche Diagnostics). Primers were designed using the Roche Probe Design 2.0 software (Roche Diagnostics) and purchased from TIB MOLBIOL (Berlin, Germany). Primer sequences, reporter probes, and qPCR conditions are given in S1 Table. All gene expression data were normalized to the housekeeping gene *RPS13* using the ΔCt method.

### Statistical analyses

Hardy-Weinberg equilibrium was tested using χ² test with one degree of freedom. Continuous variables with skewed distributions were log_*e*_-transformed prior to statistical analysis. Multiple linear regression analyses were performed using the least-squares method. For the analyses in the overall study population, the trait of interest was chosen as outcome variable and the SNP genotype (in the additive inheritance model) as independent variable. Gender, age, BMI, OGTT-derived insulin sensitivity, and lipid-lowering medication (dummy variable for drug classes) were considered as confounding variables and included wherever appropriate. To account for multiple testing (10 SNPs tested in parallel), we corrected the α-level of significance according to Bonferroni and considered a p-value <0.0051 as statistically significant. SNP associations with p-values ≥0.0051 and <0.05 were termed nominal. Associations of SNPs rs4288294 and rs116697954 (in the additive inheritance model) with the gender-dependent Framingham risk score (10-year risk categories as nominal outcome variable) were tested in 694 women and 364 men with BMI and lipid-lowering medication as confounding variables using nominal logistic regression analysis. The effects of the two SNPs (i) on HDL2- and HDL3-cholesterol in 34 subjects and (ii) on gene expression in PBMCs from 29 donors were assessed by multiple linear regression analysis. Due to very low numbers of homozygous minor allele carriers in these latter analyses, the SNPs were tested in the dominant inheritance model only (CC vs. CT+TT). Based on the strictly focused and hypothesis-driven nature of these follow-up investigations (focused on two SNPs only; all other SNPs were not followed up because they failed significance for association with HDL-cholesterol and/or apoA1 in the overall study population), we here abstained from correcting the α-level for multiple testing and considered a p-value <0.05 as statistically significant. All analyses were performed with the JMP 10.0 software (SAS Institute, Cary, NC, USA).

## Results

In this study, an overall study population of 2,068 subjects was subjected to genotyping. Of these, nearly two thirds were female (64.5%). The mean age was 39.6 years, the mean BMI 30.8 kg/m², and 69.8% of the participants displayed normal glucose tolerance whereas 12.5% had isolated impaired fasting glycaemia, 9.7% isolated impaired glucose tolerance, and 8.1% impaired fasting glycaemia and impaired glucose tolerance. The clinical characteristics of the overall study population and the major subgroups are given in Table 1.

The 10 non-linked tagging SNPs were representative for all other common genetic variants (MAF ≥0.05) in the *PIK3CG* gene locus (100% coverage) and were all found to be in Hardy-Weinberg equilibrium (p>0.09). The MAFs observed in our study population were similar to those reported for the CEU population by the 1000 Genomes Project (all differences between MAF_TÜF_ and MAF_CEU_ ≤5%).

In the association analyses, parameters of body fat content and body fat distribution were adjusted for gender and age. Blood glucose levels, insulin sensitivity measures, and adipokine and cytokine levels were adjusted for gender, age, and BMI. Insulin secretion indices were adjusted for gender, age, BMI, and insulin sensitivity, and finally, plasma lipid concentrations were adjusted for gender, age, BMI, and anti-hyperlipidaemic medication (among the 2,016 subjects analyzed for plasma lipids, 97.3% did not receive anti-hyperlipidaemic medication, 2.4% were on statins, 0.1% on fibrates, 0.1% on ezetimibe, and <0.05% on combination therapy).

**Fig 1:**
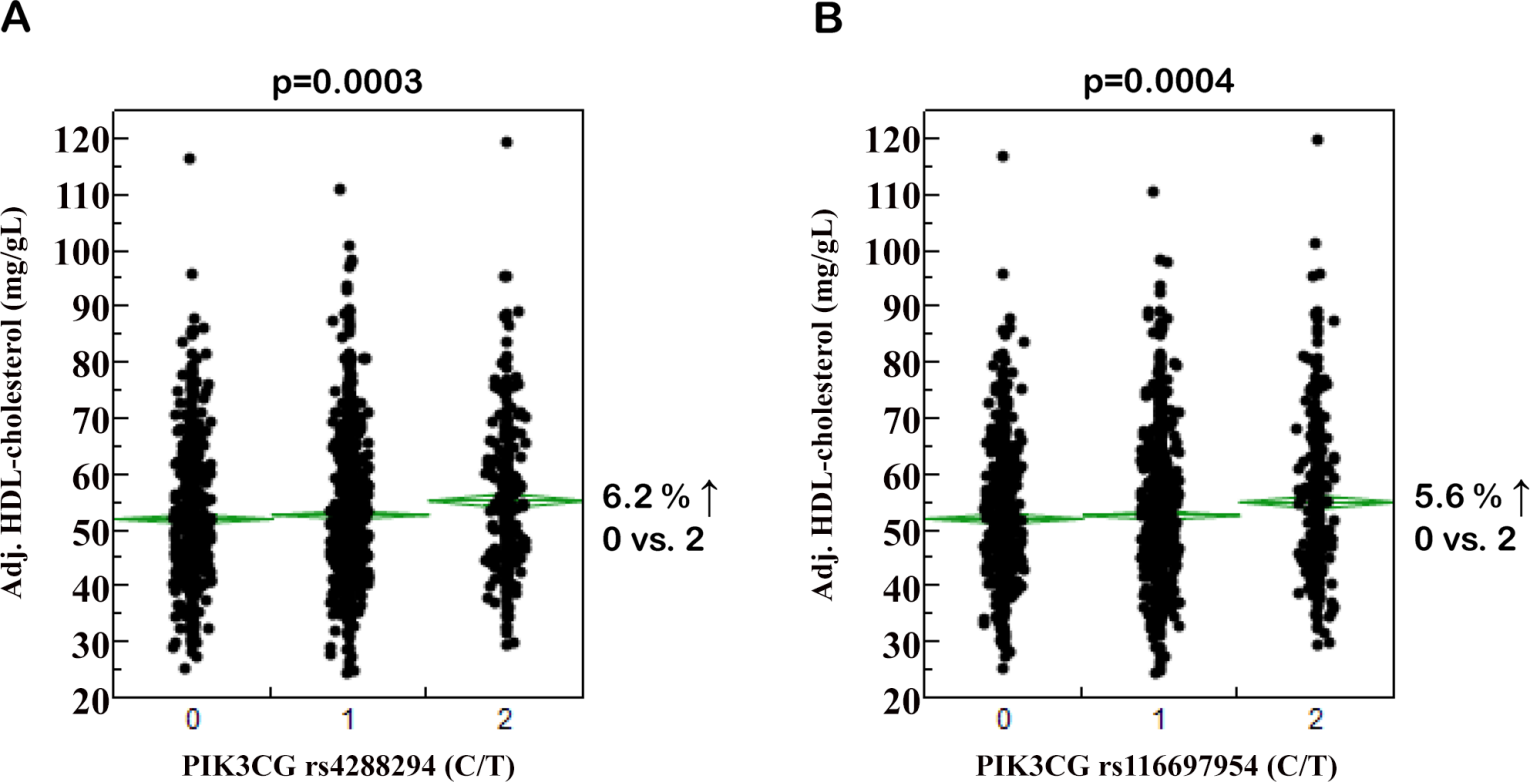
Associations of *PIK3CG* SNPs rs4288294 (A) and rs116697954 (B) with plasma HDL-cholesterol concentrations. Adjustment of plasma HDL-cholesterol concentrations (N = 2,016) was achieved by multiple linear regression modelling with gender, age, BMI, and anti-hyperlipidaemic medication as confounding variables. On the x-axes, the number of minor T-alleles is given. The SNPs were tested in the additive inheritance model. HDL–high-density lipoprotein; SNP–single nucleotide polymorphism.

After appropriate adjustment, none of the tested SNPs showed significant association with body fat content and/or distribution, adipokine or cytokine concentrations, insulin sensitivity, insulin secretion, or blood glucose concentrations (p>0.0127, all; S2–S6 Tables). However, six SNPs displayed at least nominal associations with HDL-cholesterol levels without affecting total or LDL-cholesterol, and two of them, i.e., rs4288294 and rs116697954, reached the level of study-wide significance (p = 0.0003 and p = 0.0004, respectively; unadjusted data in Table 2; adjusted data and effect sizes in Fig 1). Both SNPs are located in intron 10, separated from each other by 747 bp. Their minor T-alleles were associated with increased HDL-cholesterol levels. Notably, the minor alleles of the four nominally associated SNPs (rs3823963, rs1129293, rs2037718, and rs10216210) were associated with decreased HDL-cholesterol (Table 2). To further strengthen these results, we tested whether the six SNPs associated with HDL-cholesterol also showed association with the major apolipoprotein of HDL, i.e., apoA1. Among the two SNPs significantly associated with HDL-cholesterol, rs4288294 was nominally (p = 0.0100) and rs116697954 significantly (p = 0.0017) associated with apoA1 levels after adjustment for gender, age, BMI, and anti-hyperlipidaemic medication, and both SNPs’ minor T-alleles were associated with elevated apoA1 levels. Among the four SNPs nominally associated with HDL-cholesterol, rs2037718 and rs10216210 were also nominally associated with apoA1 concentrations (p = 0.0242 and p = 0.0127, respectively; minor alleles associated with reduced apoA1 levels); rs3823963 and rs1129293 did neither reveal significant nor nominal associations with apoA1 (p≥0.1, both). These results reflect our SNP results with HDL-cholesterol.

**Table 2:** Associations of *PIK3CG* tagging SNPs with plasma lipid concentrations (N_OGTT_ = 2,016).

	Genotype	N_OGTT_	FFA, fasting (μmol/L)	Triglycerides, fasting (mg/dL)	Total cholesterol, fasting (mg/dL)	LDL-cholesterol, fasting (mg/dL)	HDL-cholesterol, fasting (mg/dL)
rs4727666	AA	1,241	593 ±242	117 ±78	191 ±37	119 ±33	54.1 ±14.5
	AG	604	597 ±261	121 ±71	192 ±36	120 ±33	53.1 ±13.5
	GG	92	577 ±205	138 ±107	200 ±35	124 ±30	52.6 ±14.2
p	-	-	0.6	**0.0255**	0.08	**0.0418**	0.1
rs3823963	TT	671	601 ±283	121 ±78	193 ±37	121 ±33	54.4 ±14.3
	TA	952	590 ±221	118 ±73	191 ±36	119 ±33	53.7 ±14.2
	AA	310	595 ±240	123 ±93	191 ±35	119 ±32	52.7 ±14.0
p	-	-	0.8	0.8	0.2	0.5	**0.0081**
rs1129293	CC	927	600 ±265	121 ±77	193 ±37	120 ±34	54.1 ±14.2
	CT	831	589 ±228	118 ±74	191 ±36	119 ±32	53.6 ±14.4
	TT	176	589 ±228	122 ±100	189 ±36	118 ±33	52.4 ±13.1
p	-	-	0.9	0.6	0.2	0.6	**0.0291**
rs17401277	CC	1,765	596 ±251	120 ±77	192 ±36	120 ±33	53.6 ±14.1
	CT	193	574 ±213	113 ±66	189 ±35	116 ±31	54.3 ±14.3
	TT	8	514 ±210	202 ±267	238 ±58	142 ±40	57.0 ±16.6
p	-	-	0.1	1.0	0.9	0.5	0.8
rs59813697	AA	1,574	593 ±250	119 ±79	192 ±37	120 ±33	53.8 ±14.1
	AC	354	599 ±236	120 ±73	191 ±35	118 ±31	53.3 ±14.4
	CC	21	599 ±221	142 ±109	205 ±40	134 ±42	52.8 ±13.3
p	-	-	0.5	0.8	0.9	0.8	0.3
rs4288294	CC	730	588 ±226	124 ±85	194 ±36	121 ±32	52.9 ±13.7
	CT	965	598 ±251	117 ±73	190 ±36	118 ±33	53.6 ±14.2
	TT	300	590 ±280	116 ±71	195 ±40	122 ±36	55.9 ±15.0
p	-	-	0.7	0.09	0.9	0.4	**0.0003**
rs849405	AA	1,607	592 ±237	119 ±76	191 ±37	119 ±33	54.0 ±14.2
	AG	380	608 ±287	122 ±83	195 ±38	123 ±34	52.5 ±13.9
	GG	29	551 ±204	119 ±51	195 ±34	122 ±27	53.0 ±15.9
p	-	-	1.0	0.9	0.1	**0.0469**	0.2
rs116697954	CC	650	586 ±224	126 ±90	194 ±36	121 ±33	52.8 ±13.9
	CT	926	603 ±254	118 ±72	190 ±36	118 ±33	53.5 ±13.8
	TT	371	588 ±266	115 ±70	194 ±38	121 ±34	55.9 ±15.3
p	-	-	0.9	0.2	0.8	0.4	**0.0004**
rs2037718	CC	710	601 ±282	117 ±73	193 ±37	120 ±34	54.6 ±14.7
	CG	971	592 ±225	119 ±76	192 ±37	119 ±32	53.3 ±13.7
	GG	333	585 ±229	125 ±91	192 ±36	121 ±34	52.8 ±14.0
p	-	-	0.7	0.5	0.5	0.9	**0.0055**
rs10216210	GG	1,114	595 ±258	120 ±75	193 ±37	120 ±33	54.1 ±14.3
	GC	756	596 ±233	117 ±74	191 ±37	120 ±33	53.3 ±14.1
	CC	144	573 ±220	125 ±107	190 ±36	117 ±33	52.4 ±13.2
p	-	-	0.8	0.5	0.3	0.6	**0.0280**

Metabolic data are shown as unadjusted raw data (means ±SD). Associations between SNP genotypes (additive inheritance model) and plasma lipid concentrations were tested by multiple linear regression analyses (standard least squares method) with gender, age, BMI, and anti-hyperlipidaemic medication as covariates. Nominal associations (p<0.05) are marked by using bold fonts, significant associations (p<0.0051 after Bonferroni correction for 10 SNPs) by using bold fonts and underlining. AUC–area under the curve; BMI–body mass index; FFA–free fatty acids; HDL–high-density lipoproptein; LDL–low-density lipoprotein; OGTT- oral glucose tolerance test; SNP–single nucleotide polymorphism

For independent replication of the HDL data, we looked into the meta-analysis data for HDL-cholesterol from genome-wide association studies and Metabochip analyses (N≥94,255) made publicly available by the Global Lipids Genetics Consortium (GLGC, http://www.sph.umich.edu/csg/abecasis/public/lipids2013, [27]). Unfortunately, none of the six SNPs with at least nominal association with HDL-cholesterol in this study was depicted on the arrays used by the GLGC. Using the SNP Annotation and Proxy Search platform of the Broad Institute (http://www.broadinstitute.org/mpg/snap/ldsearch.php), we could identify proxies (with r²>0.8) depicted on the GLGC arrays for four of the six SNPs. However, none of the proxy SNPs reached the level of nominal significance in the GLGC data for association with HDL-cholesterol adjusted for gender and age (p≥0.07, all; subjects on lipid-lowering medication were excluded from the meta-analysis). Association data additionally adjusted for BMI were not available from GLGC. Performing the analyses in our study population without adjustment for BMI raised the p-value of SNP rs4288294 from 0.0003 to 0.0055 (rendering this hit only nominally associated) and of SNP rs116697954 from 0.0004 to 0.0018. Furthermore, BMI exclusion from adjustment abolished the associations of three SNPs described before as nominal hits (rs3823963, rs1129293, and rs10216210; p≥0.08, all).

From 694 female and 364 male study participants, blood pressure and cigarette consumption data were available. Therefore, we tested in these subjects whether the two significantly HDL-associated SNPs, i.e., rs4288294 and rs116697954, associate with the gender-dependent Framingham risk score for cardiovascular disease risk. After adjustment for BMI and anti-hyperlipidaemic medication, SNPs rs4288294 and rs116697954 were significantly associated with the risk score for women (p = 0.0354 and p = 0.0313, respectively; no Bonferroni correction applied in this focused follow-up investigation), with female minor T-allele carriers having a reduced cardiovascular disease risk. Neither SNP was associated with the risk score in the smaller group of men (p = 0.2, both).

**Fig 2:**
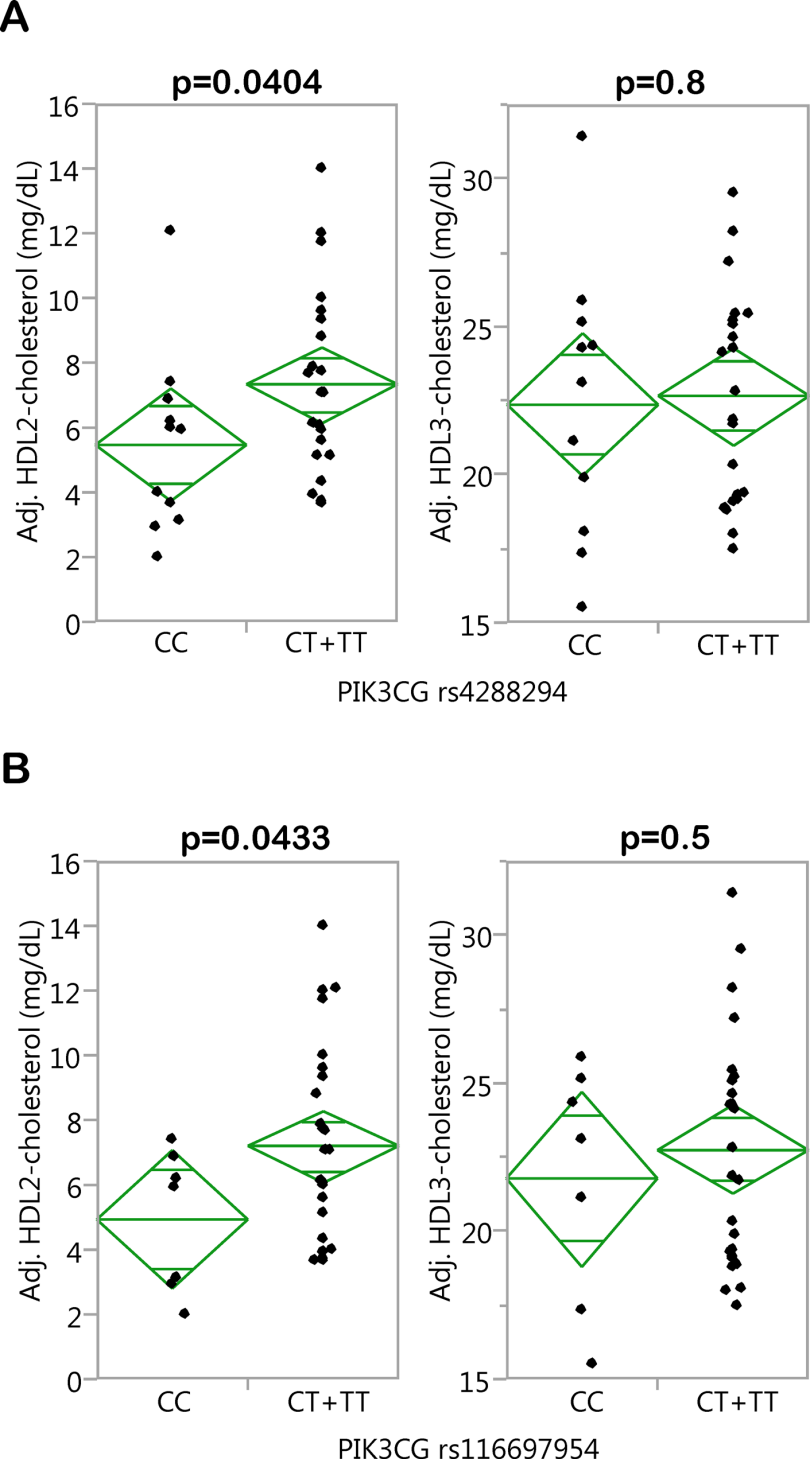
Associations of *PIK3CG* SNPs rs4288294 (A) and rs116697954 (B) with HDL2- and HDL3-cholesterol concentrations. The HDL-cholesterol subfractions HDL2 and HDL3 were obtained by ultracentrifugation. Adjustment of HDL2- and HDL3-cholesterol concentrations (N = 34) was achieved by multiple linear regression modelling with gender, age, and BMI as confounding variables. The SNPs were tested in the dominant inheritance model. HDL–high-density lipoprotein; SNP–single nucleotide polymorphism.

Then, we addressed the question whether the two SNPs affect HDL2- and/or HDL3-cholesterol concentrations. To this end, we looked into HDL fractionation data generated during an earlier investigation [19]. In this subgroup of 34 TÜF participants (clinical characteristics given in S7 Table), the minor T-alleles of SNPs rs4288294 and rs116697954 were significantly associated with higher HDL2- (p = 0.0404 and 0.0433, respectively), but not HDL3- (p = 0.8 and p = 0.5, respectively), cholesterol after adjustment for gender, age, and BMI (Fig 2). In this analysis, CT+TT were jointly analyzed (dominant inheritance model) due to the very low number of TT homozygotes (N = 5 and 7, respectively). Of the 34 participants, 15 had fatty liver (intrahepatic lipid content ≥5.5%) whereas 19 had no fatty liver. Inclusion of the presence/absence of fatty liver as a nominal confounding variable in the multiple linear regression analysis did not affect the association of SNP rs4288294 with HDL2-cholesterol (p = 0.0412; association with HDL3: p = 0.8). The association of SNP rs116697954, the SNP with the smaller effect size on HDL-cholesterol in the overall population (Fig 1B), with HDL2-cholesterol did no longer reach the level of significance (p = 0.06; association with HDL3: p = 0.5).

**Fig 3:**
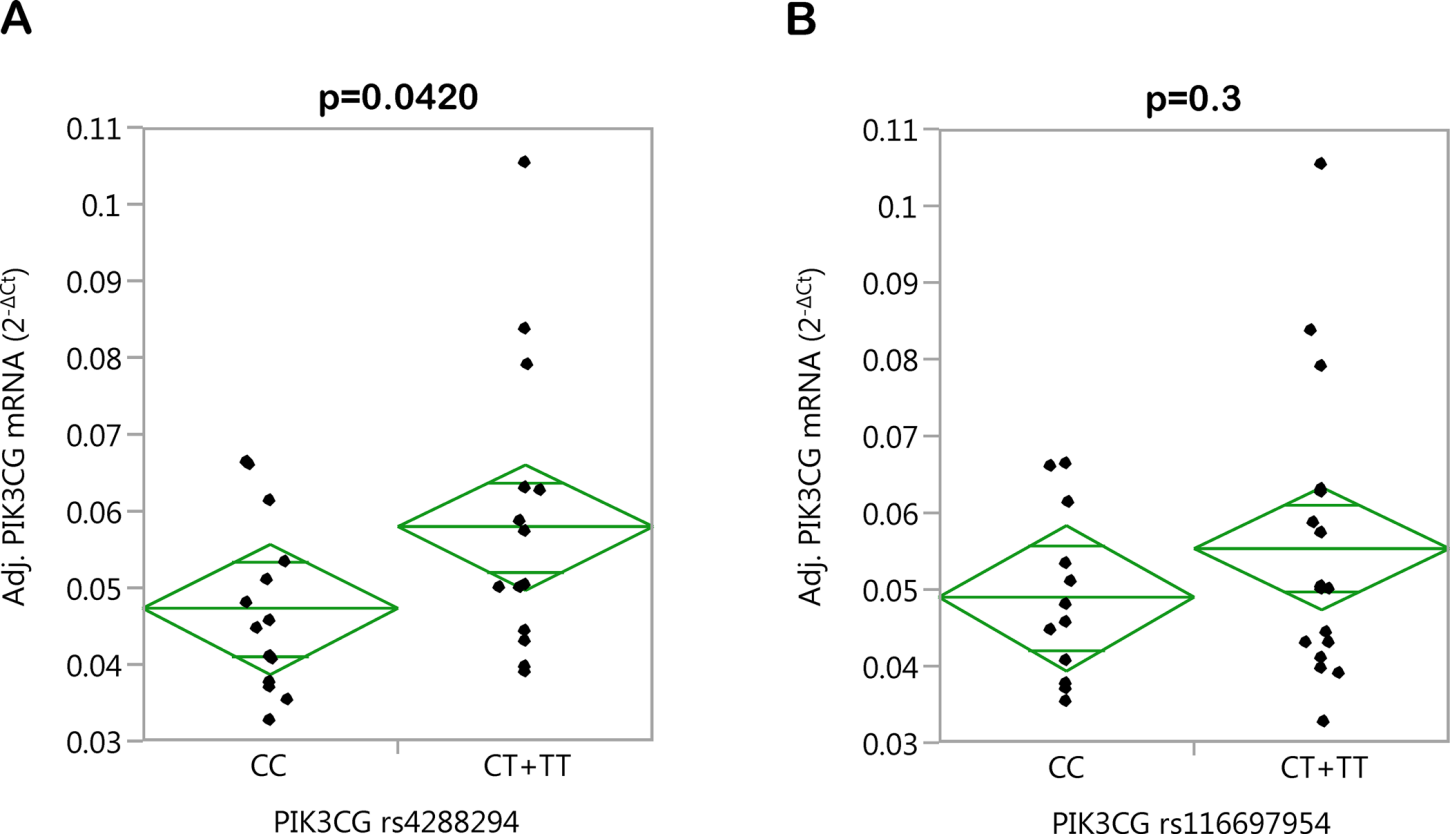
Associations of *PIK3CG* SNPs rs4288294 (A) and rs116697954 (B) with *PIK3CG* gene expression in PBMCs. After isolation of PBMCs from whole blood, the cellular *PIK3CG* mRNA content was determined by qPCR. Adjustment of *PIK3CG* mRNA expression (N = 29) was achieved by multiple linear regression modelling with gender, age, body height, and waist-to-hip ratio as confounding variables. The SNPs were tested in the dominant inheritance model. PBMCs–peripheral blood mononuclear cells; qPCR–quantitative real-time polymerase chain reaction; SNP–single nucleotide polymorphism.

In a first attempt to mechanistically understand the effects of the major HDL-associated SNPs rs4288294 and rs116697954, we measured *PIK3CG* mRNA expression in PBMCs from 29 randomly selected study participants (22 women, 7 men; age 40.9 ±12.9 years; BMI 32.1 ±4.3 kg/m²; means ±SD) and performed multiple linear regression analysis. Among the anthropometric data available, we identified age (p = 0.0070), body height (p = 0.0209), and waist-hip ratio (p = 0.0275) as significant independent determinants of *PIK3CG* gene expression explaining about 34% of the variation in *PIK3CG* mRNA levels and therefore included these parameters as covariates in the multiple linear regression model. Even though not significant, we included gender as well because of the marked gender imbalance in this subgroup (22 women, 7 men). Again, the two SNPs were tested in the dominant inheritance model due to the very low number of TT homozygotes (N = 6 and 7, respectively). Applying this regression model, we observed significantly increased *PIK3CG* gene expression in minor T-allele carriers of SNP rs4288294 (p = 0.0420; Fig 3A). The effect of SNP rs116697954, which had a smaller effect on HDL-cholesterol in the overall study population, did not pass the significance threshold (p = 0.3; Fig 3B). Even though PBMCs are probably not a major source of cholesteryl ester transfer protein (CETP; mainly expressed in liver and lymph nodes), lecitihin-cholesterol acyltransferase (LCAT; rather ubiquitously expressed), phospholipid transfer protein (PLTP; mainly expressed in thymus, retina, and lung), and scavenger receptor B1 (SRB1; encoded by the *SCARB1* gene; mainly expressed in the adrenal cortex), important proteins involved in HDL metabolism/turnover, we reasoned that if a non-coding genetic variant exerts an effect on these genes’ expression at their major expression sites this should also be reflected in cells with more moderate expression levels. Indeed, only *CETP* mRNA expression ranged at the detection limit (Cp-value >35), whereas the mRNA levels of *LCAT* (Cp-value 32.7), *PLTP* (Cp-value 32.0), and *SCARB1* (Cp-value 30.5) were reproducibly detectable in PBMCs by qPCR. However, we could not detect any significant association of the two SNPs tested, i.e., rs4288294 and rs116697954, with the expression of these genes (p>0.1, all).

## Discussion

In this work, we investigated the impact of common genetic variation in the *PIK3CG* locus, represented by 10 non-linked non-coding tagging SNPs, on inflammatory traits and inflammation-related metabolic traits in individuals at increased risk for type-2 diabetes.

Contrary to our expectations, none of the SNPs directly influenced blood inflammatory markers, such as leukocyte number, plasma CRP levels, or serum IL-6, TNF-α, or MCP-1 concentrations. Moreover, we did not observe any associations with metabolic traits like body fat content, body fat distribution, serum adipokine concentrations, plasma glucose levels, insulin sensitivity, or insulin secretion. However, we identified two SNPs with study-wide significant effects on plasma HDL-cholesterol (rs4288294 and rs116697954). Homozygous carriers of these SNPs’ minor T-alleles had 5–6% higher HDL-cholesterol levels compared to homozygous major allele carriers. Moreover, we found four additional SNPs nominally associated with HDL-cholesterol with their minor alleles being associated with reduced HDL-cholesterol. Even though not significant and with divergent effect directions, these additional findings support our suggestion that the *PIK3CG* locus affects plasma HDL-cholesterol and argue against a mere chance finding. Opposing effect directions of non-coding SNPs may have multiple reasons: e.g., one SNP in a *cis*-regulatory element, such as an enhancer structure, may strengthen the binding of a transcription factor to this element whereas another SNP within the same element–and some so-called stretch enhancers can span several kb [28]–may weaken it; or one SNP may enhance/weaken the binding of a transcriptional activator to the locus whereas another SNP may enhance/weaken the binding of a transcriptional repressor.

Based on (i) the observation that the HDL-associated *PIK3CG* SNPs identified herein were not depicted on the SNP arrays used by the GLGC, (ii) the possibility that the proxies found on the arrays may not perfectly represent our hits, and (iii) the fact that our analyses diverge from those of the GLGC meta-analysis with respect to adjustment for potential confounders, we can add a new candidate locus to the 46 HDL loci identified up to now by meta-analysis of genome-wide association studies [28]. However, this new candidate locus clearly awaits replication in larger study populations applying identical statistical analyses and adjustments of data. In this context, we could show in our study that the lack of adjustment for BMI (as was the case in the GLGC meta-analysis) abolished three of our four nominal HDL-cholesterol hits and raised the p-value of one of our two significant hits to the nominal level. Thus, adjustment for BMI appears crucial to identify associations of *PIK3CG* SNPs with HDL-cholesterol.

As a first step towards an understanding of the mechanism(s) underlying the SNPs’ association with HDL-cholesterol, we could demonstrate that rs4288294 was significantly associated with *PIK3CG* gene expression in PBMCs from 29 donors, with minor T-allele carriers displaying higher *PIK3CG* expression. Larger PBMC collections would be needed to detect a consistent significant effect in minor T-allele carriers of rs116697954 (least significant number estimated from the model: N = 83). Even though we corrected for the gender imbalance observed in our PBMC donors (22 women, 7 men) by including gender as a confounder in our multiple linear regression analyses, this adjustment could have been insufficient to completely exclude any gender bias, and larger PBMC collections with a more balanced gender distribution would allow more stringent conclusions. How the *PIK3CG* gene product PI3Kγ molecularly influences HDL formation and/or clearance is hitherto unknown, and *PIK3CG* knockout mice, available for about 15 years now, have not yet been assessed for altered HDL-cholesterol concentrations. There is one very recent interesting report providing evidence for an involvement of PI3Kγ in LDL uptake by macrophages via pinocytosis favouring foam-cell formation [29]. Whether PI3Kγ participates in HDL uptake/clearance by a similar mechanism was unfortunately not investigated, but could be a possibility. Since HDL2 is formed from HDL3, our finding that the minor T-alleles of SNPs rs4288294 and rs116697954 associate with higher HDL2-, but not HDL3-, cholesterol may point to a role of PI3Kγ in HDL clearance rather than in increased HDL biogenesis. With respect to the clinical relevance of these HDL subspecies, low HDL2 concentrations were reported to have a stronger predictive value for coronary heart disease than low HDL3 concentrations in a large prospective study [30].

For decades, high plasma HDL-cholesterol concentrations were considered as anti-atherosclerotic and cardioprotective based on a large body of epidemiological and experimental data (reviewed in [31,32]). Recently, however, a series of clinical trials aimed at increasing HDL-cholesterol by pharmacological means failed to show improvements in cardiovascular risk [33–38]. Thus, the “HDL hypothesis” that states that decreased plasma HDL concentrations lead to impaired reverse cholesterol transport thereby accelerating atherosclerosis has recently been questioned, and further investigations are needed to clarify the reasons for these treatment studies’ inefficacy. Moreover, it remains to be shown whether the mild but physiological and lifelong elevation in plasma HDL concentrations resulting from naturally occurring genetic variation (as shown here), in contrast to the artificial elevation of plasma HDL by pharmacological intervention, are beneficial for cardiovascular endpoints. Maybe, it is even more HDL functionality, particularly cholesterol efflux capacity, that contributes to the assumed anti-atherosclerotic effects. A recently published work showed a strong association between HDL-cholesterol efflux capacity and cardiovascular events [39]. This capacity, however, is only weekly associated with HDL cholesterol serum levels [39]. Certainly, studies addressing the effects of the *PIK3CG* SNPs on HDL function and turnover could help defining their contribution to atherosclerosis and cardiovascular disease. We could show here that carriers of the HDL-raising minor T-alleles of rs4288294 and rs116697954 have significantly reduced cardiovascular risk according to the Framingham risk score, at least in the larger, and thus better powered, group of women. This result however has to be interpreted cautiously as HDL-cholesterol is an integral part of the score.

In conclusion, we could demonstrate that common genetic variants in the *PIK3CG* locus (encoding PI3Kγ), possibly via effects on *PIK3CG* gene expression, determine plasma HDL-cholesterol concentrations. Since HDL2-, but not HDL3-, cholesterol is influenced by the variants, PI3Kγ may play a role in HDL clearance rather than in HDL biogenesis. Even though the molecular pathways connecting PI3Kγ and HDL metabolism remain to be further elucidated, this finding could add a novel aspect to the pathophysiological role of PI3Kγ in atherogenesis. Our results should encourage genetic studies assessing the effects of *PIK3CG* SNPs (e.g., rs4288294 and rs116697954) on cardiovascular endpoints that are not available in our study.

## Supporting Information

S1 Table Primer sequences, reporter probes, and qPCR conditions. (DOCX)

S2 Table Associations of *PIK3CG* tagging SNPs with body fat content/distribution (N_Overall_ = 2,068; N_MRI_ = 361; N_MRS_ = 481). (DOCX)

S3 Table Associations of *PIK3CG* tagging SNPs with adipokines and inflammatory parameters (N_Overall_ = 2,068; N_Cytokines_ = 383; N_Adipokines_ = 1,243). (DOCX)

S4 Table Associations of *PIK3CG* tagging SNPs with insulin sensitivity (N_OGTT_ = 2,066; N_Clamp_ = 499). (DOCX)

S5 Table Associations of *PIK3CG* tagging SNPs with insulin secretion (N_OGTT_ = 2,000; N_IVGTT_ = 306). (DOCX)

S6 Table Associations of *PIK3CG* tagging SNPs with plasma glucose concentrations (N_OGTT_ = 2,066). (DOCX)

S7 Table Clinical data of the subgroup with HDL2- and HDL3-cholesterol measurements (N = 34). (DOCX)

## Abbreviations

AIR: acute insulin response
apoA1: apolipoprotein A1
AUC: area under the curve
BMI: body mass index
CETP: cholesteryl ester transfer protein
CEU: Central European
FFA: free fatty acid
GLGC: Global Lipids Genetics Consortium
HDL: high-density lipoprotein
HEC: hyperinsulinaemic-euglycaemic clamp
HOMA-IR: homoeostasis model assessment of insulin resistance
IHL: intrahepatic lipids
IL-6: interleukin 6
ISI: insulin sensitivity index
IVGTT: intravenous glucose tolerance test
LCAT: lecithin-cholesterol acyltransferase
LDL: low-density lipoprotein
MAF: minor allele frequency
MCP-1: monocyte chemoattractant protein 1
MRI: magnetic resonance imaging
MRS: magnetic resonance spectroscopy
OGTT: oral glucose tolerance test
PBMCs: peripheral blood mononuclear cells
PI3K: phosphoinositide 3-kinase
PLTP: phospholipid transfer protein
qPCR: quantitative real-time polymerase chain reaction
SNP: single nucleotide polymorphism
SRB1: scavenger receptor B1
TAT: total adipose tissue
TNF-α: tumour necrosis factor α
TÜF: TÜbingen Family
VAT: visceral adipose tissue
