# Supplementary material for: Variation in the Phosphoinositide 3-Kinase Gamma Gene Affects Plasma HDL-Cholesterol without Modification of Metabolic or Inflammatory Markers
Source: PLoS One. 2015 Dec 10;10(12):e0144494. doi: 10.1371/journal.pone.0144494 (PMC4675530; doi:10.1371/journal.pone.0144494)
Supplement: S1 Table — (DOCX) [file pone.0144494.s001.docx]

**Table S1. Primer sequences, reporter probes, and qPCR conditions**

| Gene | Forward primer (5’-3’) | Reverse primer (5’-3’) | Fluorescent reporter probe (Universal Probe Library No.) | Amplicon length (bp) |
| --- | --- | --- | --- | --- |
| *CETP* | caagtcaagtatgggttgcaca | gacatcaatggacttggcttc | 66 | 93 |
| *LCAT* | atgctggtcttggcctca | gctgctcctctttcagcttg | 37 | 73 |
| *PIK3CG* | ggctcaaagaaaaatccccta | agcctgcacaggaataaacaa | 86 | 75 |
| *PLTP* | ccacctactttgggagcatt | cagcttcaatggggagtca | 3 | 62 |
| *RPS13* | ccccacttggttgaagttga | acaccatgtgaatctctcagga | 68 | 114 |
| *SCARB1* | catcaagcagcaggtcctta | cggagagatagaaggggattagg | 12 | 95 |

The qPCR conditions were identical for all mRNAs quantified: initial denaturation at 95°C for 5 min; followed by 45 cycles of 95°C for 10 sec, 60°C for 30 sec, and 72°C for 1 sec; followed by final cooling at 40°C for 10 sec.
