# Supplementary material for: Variation in the Phosphoinositide 3-Kinase Gamma Gene Affects Plasma HDL-Cholesterol without Modification of Metabolic or Inflammatory Markers
Source: PLoS One. 2015 Dec 10;10(12):e0144494. doi: 10.1371/journal.pone.0144494 (PMC4675530; doi:10.1371/journal.pone.0144494)
Supplement: S2 Table — (DOCX) [file pone.0144494.s002.docx]

**Table S2. Associations of *PIK3CG* tagging SNPs with body fat content/distribution (N_Overall_=2,068; N_MRI_=361; N_MRS_=481)**

|  | Genotype | N Overall | BMI (kg/m²) | Waist (cm) | Body fat content  (%) | N MRI | TAT  (% BW) | VAT (% BW) | N MRS | IHL (% signal) |
| --- | --- | --- | --- | --- | --- | --- | --- | --- | --- | --- |
| rs4727666 | AA | 1,274 | 30.7 ±9.4 | 97 ±20 | 33.4 ±12.8 | 219 | 30.4 ±9.3 | 3.41 ±1.75 | 291 | 6.38 ±6.64 |
|  | AG | 619 | 31.0 ±9.8 | 98 ±20 | 33.7 ±12.8 | 100 | 30.2 ±9.3 | 3.12 ±1.70 | 141 | 5.52 ±5.91 |
|  | GG | 94 | 30.9 ±9.0 | 98 ±17 | 32.8 ±12.9 | 15 | 33.2 ±7.0 | 3.59 ±1.36 | 20 | 8.59 ±8.76 |
| p | - | - | 0.5 | 0.6 | 1.0 | - | 0.8 | 0.4 | - | 0.9 |
| rs3823963 | TT | 682 | 31.2 ±9.9 | 98 ±20 | 33.6 ±13.0 | 114 | 31.1 ±9.4 | 3.34 ±1.81 | 152 | 5.82 ±6.35 |
|  | TA | 982 | 30.7 ±9.4 | 97 ±19 | 33.8 ±12.7 | 163 | 29.9 ±9.3 | 3.34 ±1.72 | 229 | 6.53 ±6.71 |
|  | AA | 318 | 30.5 ±9.3 | 98 ±21 | 32.5 ±12.7 | 57 | 30.7 ±8.4 | 3.33 ±1.54 | 71 | 6.19 ±6.51 |
| p | - | - | 0.2 | 0.4 | 0.4 | - | 0.9 | 0.6 | - | 0.5 |
| rs1129293 | CC | 948 | 31.1 ±9.9 | 98 ±20 | 33.9 ±13.1 | 154 | 30.3 ±9.0 | 3.33 ±1.76 | 209 | 6.33 ±6.68 |
|  | CT | 854 | 30.6 ±9.2 | 97 ±19 | 33.4 ±12.5 | 146 | 31.0 ±9.5 | 3.31 ±1.67 | 200 | 6.27 ±6.57 |
|  | TT | 182 | 29.8 ±8.9 | 96 ±20 | 32.0 ±12.9 | 34 | 28.8 ±8.3 | 3.51 ±1.77 | 43 | 5.64 ±5.94 |
| p | - | - | 0.1 | 0.1 | 0.1 | - | 0.8 | 0.5 | - | 0.8 |
| rs17401277 | CC | 1,812 | 30.9 ±9.5 | 97 ±20 | 33.6 ±12.8 | 308 | 30.8 ±8.9 | 3.33 ±1.71 | 415 | 6.30 ±6.70 |
|  | CT | 196 | 30.1 ±9.4 | 97 ±21 | 32.1 ±12.8 | 31 | 27.8 ±11.1 | 3.27 ±1.79 | 42 | 5.64 ±4.92 |
|  | TT | 8 | 33.1 ±9.5 | 104 ±19 | 38.1 ±10.8 | 2 | 27.3 ±4.0 | 4.07 ±2.33 | 2 | 2.84 ±1.87 |
| p | - | - | 0.4 | 1.0 | 0.06 | - | **0.0127** | 0.8 | - | 0.7 |
| rs59813697 | AA | 1,613 | 30.7 ±9.4 | 97 ±19 | 33.4 ±12.8 | 275 | 30.6 ±9.4 | 3.36 ±1.77 | 372 | 5.95 ±6.34 |
|  | AC | 364 | 31.3 ±10.3 | 99 ±21 | 34.2 ±13.1 | 62 | 30.2 ±8.4 | 3.26 ±1.44 | 82 | 7.64 ±7.40 |
|  | CC | 22 | 29.1 ±6.1 | 96 ±16 | 31.6 ±12.1 | 1 | 31.0 | 2.10 | 2 | 2.81 ±1.20 |
| p | - | - | 0.7 | 0.3 | 0.4 | - | 1.0 | 0.8 | - | 0.2 |

(continued on next page)

|  | Genotype | N Overall | BMI (kg/m²) | Waist (cm) | Body fat content  (%) | N MRI | TAT  (% BW) | VAT (% BW) | N MRS | IHL (% signal) |
| --- | --- | --- | --- | --- | --- | --- | --- | --- | --- | --- |
| rs4288294 | CC | 748 | 30.5 ±9.3 | 97 ±20 | 32.9 ±12.7 | 121 | 30.4 ±8.6 | 3.35 ±1.58 | 166 | 6.24 ±6.67 |
|  | CT | 994 | 30.8 ±9.5 | 97 ±19 | 33.7 ±12.7 | 184 | 30.3 ±9.3 | 3.20 ±1.71 | 241 | 6.31 ±6.56 |
|  | TT | 302 | 31.1 ±9.6 | 98 ±20 | 33.9 ±12.9 | 51 | 31.6 ±10.0 | 3.66 ±1.92 | 70 | 5.92 ±6.79 |
| p | - | - | 0.3 | 0.5 | 0.2 | - | 1.0 | 0.7 | - | 0.8 |
| rs849405 | AA | 1,646 | 30.7 ±9.4 | 97 ±20 | 33.4 ±12.8 | 292 | 30.3 ±9.2 | 3.34 ±1.69 | 383 | 6.29 ±6.67 |
|  | AG | 392 | 31.5 ±10.2 | 99 ±20 | 33.8 ±12.7 | 64 | 31.0 ±9.0 | 3.27 ±1.79 | 91 | 5.64 ±6.23 |
|  | GG | 30 | 29.7 ±7.1 | 96 ±15 | 31.8 ±12.8 | 5 | 34.2 ±8.6 | 3.89 ±1.78 | 7 | 8.94 ±7.35 |
| p | - | - | 0.3 | 0.2 | 0.5 | - | 0.3 | 0.9 | - | 0.9 |
| rs116697954 | CC | 663 | 30.6 ±9.3 | 97 ±20 | 33.0 ±12.7 | 105 | 30.1 ±8.5 | 3.43 ±1.66 | 149 | 6.37 ±6.74 |
|  | CT | 959 | 31.0 ±9.6 | 98 ±20 | 33.9 ±12.9 | 175 | 30.8 ±9.3 | 3.18 ±1.65 | 227 | 6.46 ±6.61 |
|  | TT | 375 | 30.6 ±9.4 | 97 ±20 | 33.1 ±12.7 | 59 | 30.6 ±9.9 | 3.61 ±1.96 | 81 | 5.32 ±6.04 |
| p | - | - | 0.8 | 1.0 | 0.8 | - | 0.6 | 0.6 | - | 0.3 |
| rs2037718 | CC | 723 | 31.1 ±9.8 | 98 ±20 | 33.6 ±12.9 | 126 | 31.3 ±9.7 | 3.36 ±1.81 | 165 | 5.65 ±6.17 |
|  | CG | 1,005 | 30.6 ±9.2 | 97 ±19 | 33.6 ±12.7 | 178 | 30.0 ±9.1 | 3.28 ±1.67 | 243 | 6.50 ±6.93 |
|  | GG | 338 | 30.8 ±9.5 | 98 ±21 | 33.0 ±12.7 | 56 | 30.1 ±8.2 | 3.48 ±1.60 | 72 | 6.55 ±6.42 |
| p | - | - | 0.4 | 0.5 | 0.7 | - | 0.9 | 1.0 | - | 0.3 |
| rs10216210 | GG | 1,139 | 30.9 ±9.7 | 98 ±20 | 33.6 ±12.9 | 192 | 30.5 ±9.3 | 3.37 ±1.74 | 252 | 6.19 ±6.58 |
|  | GC | 782 | 30.9 ±9.4 | 98 ±20 | 33.7 ±12.6 | 142 | 30.9 ±9.4 | 3.24 ±1.62 | 194 | 6.35 ±6.80 |
|  | CC | 145 | 29.3 ±8.5 | 95 ±19 | 31.3 ±12.6 | 26 | 27.6 ±7.0 | 3.61 ±1.94 | 34 | 5.63 ±5.68 |
| p | - | - | 0.2 | 0.2 | 0.3 | - | 0.8 | 1.0 | - | 1.0 |

Metabolic data are shown as unadjusted raw data (means ±SD). Associations between SNP genotypes (additive inheritance model) and body fat content/distribution were tested by multiple linear regression analyses (standard least squares method) with gender and age as covariates. Nominal associations (p<0.05) are marked by using bold fonts. Bioimp – bioelectrical impedance; BMI – body mass index; BW – body weight; IHL – intrahepatic lipids; MRI – magnetic resonance imaging; MRS – magnetic resonance spectroscopy; SNP – single nucleotide polymorphism; TAT – total adipose tissue; VAT – visceral adipose tissue
