# Supplementary material for: Variation in the Phosphoinositide 3-Kinase Gamma Gene Affects Plasma HDL-Cholesterol without Modification of Metabolic or Inflammatory Markers
Source: PLoS One. 2015 Dec 10;10(12):e0144494. doi: 10.1371/journal.pone.0144494 (PMC4675530; doi:10.1371/journal.pone.0144494)
Supplement: S3 Table — (DOCX) [file pone.0144494.s003.docx]

**Table S3. Associations of *PIK3CG* tagging SNPs with adipokines and inflammatory parameters (N_Overall_=2,068; N_Cytokines_=383; N_Adipokines_=1,243)**

|  | Genotype | N Overall | Leukocytes, fasting (µl^-1^) | CRP, fasting  (mg/dL) | N Cytokines | IL-6  (pg/mL) | TNF-α  (pg/mL) | MCP-1 (pg/mL) | N Adipokines | Leptin (ng/mL) | Adiponectin (µg/mL) |
| --- | --- | --- | --- | --- | --- | --- | --- | --- | --- | --- | --- |
| rs4727666 | AA | 1,274 | 6,595 ±1,891 | 0.373 ±0.513 | 242 | 0.875 ±0.892 | 2.82 ±5.20 | 177 ±83 | 771 | 27.8 ±31.2 | 14.6 ±7.4 |
|  | AG | 619 | 6,652 ±1,925 | 0.395 ±0.562 | 101 | 0.901 ±0.720 | 2.51 ±5.11 | 194 ±113 | 363 | 27.5 ±31.9 | 14.4 ±7.4 |
|  | GG | 94 | 6,406 ±1,606 | 0.386 ±0.458 | 12 | 0.998 ±1.176 | 2.50 ±3.41 | 158 ±79 | 54 | 29.2 ±25.7 | 14.1 ±8.2 |
| p | - | - | 0.8 | 0.3 | - | 0.7 | 0.9 | 0.8 | - | 0.2 | 0.2 |
| rs3823963 | TT | 682 | 6,651 ±1,930 | 0.419 ±0.565 | 125 | 0.879 ±0.861 | 2.81 ±5.83 | 178 ±104 | 406 | 27.4 ±29.8 | 14.7 ±7.8 |
|  | TA | 982 | 6,587 ±1,869 | 0.356 ±0.492 | 168 | 0.870 ±0.825 | 2.51 ±3.64 | 181 ±85 | 581 | 28.0 ±30.9 | 14.5 ±7.4 |
|  | AA | 318 | 6,547 ±1,863 | 0.379 ±0.525 | 62 | 0.952 ±0.936 | 3.13 ±6.80 | 185 ±88 | 199 | 28.6 ±34.8 | 14.1 ±6.7 |
| p | - | - | 1.0 | 0.6 | - | 1.0 | 0.6 | 0.6 | - | 0.7 | 0.8 |
| rs1129293 | CC | 948 | 6,594 ±1,881 | 0.407 ±0.543 | 165 | 0.883 ±0.887 | 2.64 ±5.40 | 177 ±99 | 559 | 27.1 ±30.6 | 14.6 ±7.7 |
|  | CT | 854 | 6,628 ±1,921 | 0.354 ±0.504 | 152 | 0.859 ±0.753 | 2.68 ±4.16 | 187 ±89 | 513 | 28.3 ±31.3 | 14.6 ±7.3 |
|  | TT | 182 | 6,477 ±1,780 | 0.361 ±0.491 | 36 | 0.983 ±1.101 | 3.42 ±7.28 | 166 ±74 | 115 | 28.7 ±33.7 | 13.3 ±5.9 |
| p | - | - | 0.5 | 0.6 | - | 1.0 | 0.1 | 0.6 | - | 0.2 | 0.5 |
| rs17401277 | CC | 1,812 | 6,610 ±1,904 | 0.383 ±0.518 | 321 | 0.907 ±0.884 | 2.73 ±5.26 | 178 ±89 | 1,082 | 27.9 ±31.6 | 14.4 ±7.5 |
|  | CT | 196 | 6,550 ±1,806 | 0.367 ±0.596 | 40 | 0.717 ±0.503 | 2.45 ±3.25 | 194 ±112 | 122 | 27.3 ±29.4 | 14.5 ±6.3 |
|  | TT | 8 | 6,706 ±1,550 | 0.326 ±0.378 | 2 | 0.435 ±0.276 | 0.76 ±0.37 | 246 ±104 | 6 | 24.6 ±15.9 | 17.0 ±8.6 |
| p | - | - | 0.9 | 0.3 | - | 0.7 | 0.6 | 0.2 | - | 0.9 | 0.7 |
| rs59813697 | AA | 1,613 | 6,625 ±1,913 | 0.383 ±0.531 | 290 | 0.879 ±0.852 | 2.80 ±5.16 | 178 ±93 | 971 | 28.2 ±31.2 | 14.5 ±7.5 |
|  | AC | 364 | 6,516 ±1,791 | 0.367 ±0.496 | 65 | 0.915 ±0.874 | 2.37 ±4.87 | 193 ±92 | 212 | 26.7 ±31.6 | 14.2 ±6.7 |
|  | CC | 22 | 6,512 ±2,006 | 0.530 ±0.656 | 2 | 1.020 ±0.792 | 1.76 ±0.37 | 126 ±64 | 15 | 27.5 ±39.7 | 16.6 ±12.9 |
| p | - | - | 0.4 | 1.0 | - | 0.8 | 0.2 | 0.8 | - | 0.1 | 0.7 |

(continued on next page)

|  | Genotype | N Overall | Leukocytes (µl^-1^) | CRP  (mg/dL) | N Cytokines | IL-6  (pg/mL) | TNF-α  (pg/mL) | MCP-1 (pg/mL) | N Adipokines | Leptin (ng/mL) | Adiponectin (µg/mL) |
| --- | --- | --- | --- | --- | --- | --- | --- | --- | --- | --- | --- |
| rs4288294 | CC | 748 | 6,525 ±1,817 | 0.363 ±0.501 | 129 | 0.942 ±0.895 | 2.59 ±5.13 | 183 ±85 | 452 | 27.6 ±32.4 | 14.1 ±7.2 |
|  | CT | 994 | 6,608 ±1,899 | 0.379 ±0.520 | 188 | 0.835 ±0.756 | 2.64 ±4.66 | 187 ±101 | 586 | 27.7 ±30.4 | 14.6 ±7.3 |
|  | TT | 302 | 6,734 ±2,017 | 0.412 ±0.575 | 60 | 0.922 ±0.988 | 2.73 ±5.68 | 156 ±69 | 191 | 27.3 ±30.2 | 14.9 ±8.1 |
| p | - | - | 0.5 | 1.0 | - | 0.9 | 0.5 | 0.3 | - | 0.1 | 0.1 |
| rs849405 | AA | 1,646 | 6,598 ±1,869 | 0.374 ±0.525 | 307 | 0.886 ±0.853 | 2.94 ±5.88 | 179 ±85 | 1,002 | 27.4 ±30.9 | 14.5 ±7.4 |
|  | AG | 392 | 6,635 ±1,973 | 0.402 ±0.511 | 73 | 0.879 ±0.802 | 2.00 ±2.82 | 196 ±115 | 225 | 30.2 ±34.0 | 13.6 ±7.2 |
|  | GG | 30 | 6,212 ±1,514 | 0.378 ±0.537 | 3 | 0.473 ±0.277 | 1.54 ±0.78 | 134 ±32 | 16 | 32.9 ±27.3 | 17.5 ±9.3 |
| p | - | - | 0.4 | 0.8 | - | 0.5 | 0.5 | 0.8 | - | **0.0160** | 0.5 |
| rs116697954 | CC | 663 | 6,531 ±1,793 | 0.369 ±0.511 | 106 | 0.926 ±0.935 | 2.66 ±5.43 | 180 ±83 | 399 | 27.6 ±31.7 | 14.3 ±7.1 |
|  | CT | 959 | 6,622 ±1,925 | 0.387 ±0.535 | 176 | 0.879 ±0.776 | 2.50 ±4.43 | 188 ±106 | 568 | 28.6 ±32.2 | 14.5 ±7.4 |
|  | TT | 375 | 6,667 ±1,953 | 0.385 ±0.513 | 76 | 0.866 ±0.930 | 3.29 ±6.02 | 162 ±67 | 228 | 26.3 ±27.3 | 14.7 ±7.7 |
| p | - | - | 0.6 | 0.6 | - | 1.0 | 0.7 | 0.6 | - | 0.2 | 0.5 |
| rs2037718 | CC | 723 | 6,667 ±1,988 | 0.405 ±0.540 | 147 | 0.814 ±0.759 | 2.93 ±5.94 | 179 ±99 | 433 | 28.4 ±31.4 | 14.6 ±7.7 |
|  | CG | 1,005 | 6,551 ±1,833 | 0.363 ±0.505 | 179 | 0.932 ±0.877 | 2.45 ±4.27 | 187 ±89 | 597 | 27.2 ±30.4 | 14.4 ±7.3 |
|  | GG | 338 | 6,596 ±1,812 | 0.376 ±0.533 | 57 | 0.897 ±0.922 | 3.22 ±7.04 | 175 ±77 | 211 | 28.9 ±34.4 | 14.1 ±6.9 |
| p | - | - | 0.7 | 0.9 | - | 0.5 | 0.7 | 0.9 | - | 0.6 | 0.9 |
| rs10216210 | GG | 1,139 | 6,593 ±1,881 | 0.395 ±0.527 | 218 | 0.861 ±0.828 | 2.91 ±5.99 | 180 ±96 | 677 | 27.4 ±30.9 | 14.4 ±7.6 |
|  | GC | 782 | 6,638 ±1,930 | 0.360 ±0.517 | 138 | 0.913 ±0.785 | 2.29 ±3.36 | 187 ±87 | 476 | 29.1 ±32.0 | 14.6 ±7.2 |
|  | CC | 145 | 6,440 ±1,672 | 0.349 ±0.496 | 27 | 0.883 ±1.179 | 3.78 ±8.29 | 165 ±77 | 89 | 26.0 ±32.9 | 13.6 ±6.1 |
| p | - | - | 0.5 | 0.6 | - | 0.8 | 0.6 | 0.9 | - | 0.3 | 1.0 |

Metabolic data are shown as unadjusted raw data (means ±SD). Associations between SNP genotypes (additive inheritance model) and adipokines/inflammatory parameters were tested by multiple linear regression analyses (standard least squares method) with gender, age, and BMI as covariates. Nominal associations (p<0.05) are marked by using bold fonts. BMI – body mass index; CRP – C-reactive protein; IL – interleukin; MCP – monocyte chemoattractant protein; SNP – single nucleotide polymorphism; TNF – tumour necrosis factor
