# Supplementary material for: Variation in the Phosphoinositide 3-Kinase Gamma Gene Affects Plasma HDL-Cholesterol without Modification of Metabolic or Inflammatory Markers
Source: PLoS One. 2015 Dec 10;10(12):e0144494. doi: 10.1371/journal.pone.0144494 (PMC4675530; doi:10.1371/journal.pone.0144494)
Supplement: S4 Table — (DOCX) [file pone.0144494.s004.docx]

**Table S4. Associations of *PIK3CG* tagging SNPs with insulin sensitivity (N_OGTT_=2,066; N_Clamp_=499)**

|  | Genotype | N OGTT | Insulin, fasting (pmol/L) | HOMA-IR (10^-6^ mol*U*L^-2^) | ISI-OGTT (10^19^ L^2^*mol^-2^) | N HEC | ISI-HEC  (10^6^ L*kg^-1^*min^-1^) |
| --- | --- | --- | --- | --- | --- | --- | --- |
| rs4727666 | AA | 1,274 | 73.0 ±61.1 | 2.86 ±2.55 | 14.7 ±10.3 | 305 | 0.084 ±0.049 |
|  | AG | 617 | 82.6 ±81.1 | 3.27 ±3.46 | 14.4 ±10.4 | 131 | 0.090 ±0.060 |
|  | GG | 94 | 78.2 ±71.7 | 3.09 ±3.05 | 14.3 ±9.6 | 29 | 0.085 ±0.058 |
| p | - | - | **0.0307** | **0.0470** | 0.3 | - | 0.1 |
| rs3823963 | TT | 680 | 78.6 ±70.9 | 3.10 ±3.01 | 14.2 ±10.0 | 168 | 0.093 ±0.060 |
|  | TA | 982 | 76.4 ±69.3 | 3.02 ±2.93 | 14.5 ±10.3 | 215 | 0.081 ±0.049 |
|  | AA | 318 | 72.0 ±61.6 | 2.80 ±2.58 | 15.4 ±11.1 | 83 | 0.083 ±0.047 |
| p | - | - | 0.3 | 0.2 | 0.3 | - | **0.0145** |
| rs1129293 | CC | 946 | 77.2 ±68.2 | 3.04 ±2.88 | 14.2 ±9.8 | 226 | 0.091 ±0.058 |
|  | CT | 854 | 76.7 ±71.2 | 3.03 ±3.01 | 14.8 ±10.6 | 188 | 0.080 ±0.047 |
|  | TT | 182 | 68.5 ±56.1 | 2.65 ±2.36 | 15.7 ±11.5 | 53 | 0.085 ±0.051 |
| p | - | - | 0.5 | 0.4 | 0.5 | - | 0.05 |
| rs17401277 | CC | 1,810 | 75.8 ±66.2 | 2.98 ±2.77 | 14.6 ±10.3 | 439 | 0.084 ±0.052 |
|  | CT | 196 | 78.1 ±83.5 | 3.10 ±3.73 | 15.0 ±10.6 | 38 | 0.099 ±0.059 |
|  | TT | 8 | 107.1 ±111.2 | 4.30 ±4.58 | 11.0 ±8.2 | - | - |
| p | - | - | 0.3 | 0.4 | 0.3 | - | 0.4 |
| rs59813697 | AA | 1,611 | 76.5 ±69.6 | 3.01 ±2.95 | 14.6 ±10.4 | 385 | 0.086 ±0.054 |
|  | AC | 364 | 75.5 ±63.5 | 2.97 ±2.67 | 14.4 ±10.0 | 79 | 0.081 ±0.049 |
|  | CC | 22 | 72.3 ±66.8 | 2.78 ±2.67 | 13.9 ±7.4 | 5 | 0.090 ±0.025 |
| p | - | - | 0.5 | 0.4 | 0.6 | - | 0.3 |

(contiuned on next page)

|  | Genotype | N OGTT | Insulin, fasting (pmol/L) | HOMA-IR (10^-6^ mol*U*L^-2^) | ISI-OGTT (10^15^ L^2^*mol^-2^) | N HEC | ISI-HEC  (10^6^ L*kg^-1^*min^-1^) |
| --- | --- | --- | --- | --- | --- | --- | --- |
| rs4288294 | CC | 748 | 75.6 ±71.1 | 2.98 ±3.02 | 15.0 ±10.6 | 184 | 0.083 ±0.048 |
|  | CT | 992 | 77.0 ±68.5 | 3.04 ±2.92 | 14.3 ±10.0 | 234 | 0.084 ±0.056 |
|  | TT | 302 | 72.9 ±56.4 | 2.86 ±2.31 | 14.4 ±10.1 | 74 | 0.092 ±0.052 |
| p | - | - | 0.7 | 0.9 | 0.8 | - | 0.3 |
| rs849405 | AA | 1,645 | 74.5 ±64.6 | 2.93 ±2.72 | 14.6 ±10.2 | 396 | 0.083 ±0.050 |
|  | AG | 391 | 82.6 ±81.0 | 3.27 ±3.47 | 14.4 ±10.3 | 93 | 0.091 ±0.062 |
|  | GG | 30 | 72.5 ±48.9 | 2.86 ±2.21 | 15.1 ±10.6 | 10 | 0.091 ±0.044 |
| p | - | - | 0.3 | 0.4 | 0.9 | - | **0.0214** |
| rs116697954 | CC | 663 | 75.7 ±71.0 | 2.97 ±2.98 | 14.8 ±10.5 | 155 | 0.084 ±0.051 |
|  | CT | 957 | 78.6 ±71.2 | 3.11 ±3.04 | 14.4 ±10.3 | 224 | 0.084 ±0.054 |
|  | TT | 375 | 71.0 ±55.4 | 2.78 ±2.28 | 14.7 ±10.1 | 94 | 0.091 ±0.055 |
| p | - | - | 0.6 | 0.9 | 0.9 | - | 0.6 |
| rs2037718 | CC | 721 | 77.7 ±69.4 | 3.07 ±2.94 | 14.5 ±10.3 | 187 | 0.090 ±0.056 |
|  | CG | 1,005 | 75.5 ±67.3 | 2.98 ±2.85 | 14.4 ±10.1 | 223 | 0.080 ±0.050 |
|  | GG | 338 | 73.8 ±66.4 | 2.88 ±2.77 | 15.0 ±10.6 | 88 | 0.085 ±0.049 |
| p | - | - | 0.5 | 0.4 | 0.6 | - | 0.2 |
| rs10216210 | GG | 1,137 | 76.6 ±67.0 | 3.02 ±2.84 | 14.3 ±9.9 | 283 | 0.088 ±0.057 |
|  | GC | 782 | 76.4 ±70.7 | 3.01 ±2.98 | 14.6 ±10.3 | 171 | 0.077 ±0.044 |
|  | CC | 145 | 67.1 ±56.2 | 2.58 ±2.36 | 16.2 ±11.9 | 45 | 0.091 ±0.054 |
| p | - | - | 0.4 | 0.3 | 0.4 | - | 0.2 |

Metabolic data are shown as unadjusted raw data (means ±SD). Associations between SNP genotypes (additive inheritance model) and insulin sensitivity were tested by multiple linear regression analyses (standard least squares method) with gender, age, and BMI as covariates. Nominal associations (p<0.05) are marked by using bold fonts. BMI – body mass index; HEC – hyperinsulinaemic-euglycaemic clamp; HOMA-IR – homoeostasis model assessment of insulin resistance; ISI – insulin sensitivity index; OGTT – oral glucose tolerance test; SNP – single nucleotide polymorphism
