# Supplementary material for: Variation in the Phosphoinositide 3-Kinase Gamma Gene Affects Plasma HDL-Cholesterol without Modification of Metabolic or Inflammatory Markers
Source: PLoS One. 2015 Dec 10;10(12):e0144494. doi: 10.1371/journal.pone.0144494 (PMC4675530; doi:10.1371/journal.pone.0144494)
Supplement: S5 Table — (DOCX) [file pone.0144494.s005.docx]

**Table S5. Associations of *PIK3CG* tagging SNPs with insulin secretion (N_OGTT_=2,000; N_IVGTT_=306)**

|  | Genotype | N OGTT | AUC_0-30 min_ Insulin/ AUC_0-30 min_ Glucose  (10^-9^) | AUC_0-120 min_ C-Peptide/ AUC_0-120 min_ Glucose  (10^-9^) | N IVGTT | AIR (pmol/L) |
| --- | --- | --- | --- | --- | --- | --- |
| rs4727666 | AA | 1,232 | 47.4 ±34.7 | 314 ±102 | 184 | 927 ±575 |
|  | AG | 597 | 48.2 ±36.4 | 314 ±102 | 82 | 928 ±673 |
|  | GG | 92 | 46.9 ±32.7 | 323 ±109 | 15 | 928 ±725 |
| p | - | - | 0.2 | 0.9 | - | 0.2 |
| rs3823963 | TT | 661 | 48.3 ±34.1 | 314 ±103 | 95 | 921 ±573 |
|  | TA | 946 | 47.6 ±35.8 | 315 ±103 | 146 | 933 ±641 |
|  | AA | 308 | 46.7 ±35.1 | 313 ±97 | 41 | 938 ±589 |
| p | - | - | 0.2 | 0.1 | - | 0.6 |
| rs1129293 | CC | 918 | 47.8 ±34.2 | 315 ±105 | 130 | 891 ±585 |
|  | CT | 823 | 48.0 ±36.5 | 315 ±100 | 127 | 990 ±650 |
|  | TT | 176 | 45.0 ±33.3 | 309 ±97 | 25 | 806 ±508 |
| p | - | - | 0.2 | 0.3 | - | 0.6 |
| rs17401277 | CC | 1,749 | 47.0 ±34.1 | 312 ±99 | 268 | 943 ±614 |
|  | CT | 192 | 52.0 ±41.0 | 329 ±125 | 20 | 857 ±648 |
|  | TT | 8 | 55.4 ±43.7 | 319 ±91 | - | - |
| p | - | - | 0.1 | 0.2 | - | 1.0 |
| rs59813697 | AA | 1,559 | 47.6 ±35.0 | 313 ±102 | 235 | 937 ±604 |
|  | AC | 351 | 47.6 ±35.4 | 316 ±104 | 49 | 907 ±656 |
|  | CC | 22 | 47.4 ±34.3 | 332 ±107 | 1 | 1,120 |
| p | - | - | 0.8 | 0.3 | - | 0.9 |

(contiuned on next page)

|  | Genotype | N OGTT | AUC_0-30 min_ Insulin/ AUC_0-30 min_ Glucose  (10^-9^) | AUC_0-120 min_ C-Peptide/ AUC_0-120 min_ Glucose  (10^-9^) | N IVGTT | AIR (pmol/L) |
| --- | --- | --- | --- | --- | --- | --- |
| rs4288294 | CC | 722 | 46.3 ±35.0 | 314 ±100 | 101 | 889 ±624 |
|  | CT | 963 | 48.0 ±35.1 | 314 ±101 | 159 | 981 ±653 |
|  | TT | 293 | 46.9 ±32.9 | 310 ±105 | 40 | 878 ±474 |
| p | - | - | 0.3 | 0.08 | - | 0.4 |
| rs849405 | AA | 1,589 | 46.9 ±34.2 | 313 ±111 | 244 | 941 ±634 |
|  | AG | 382 | 49.2 ±36.4 | 318 ±103 | 56 | 916 ±527 |
|  | GG | 29 | 50.1 ±37.2 | 323 ±89 | 6 | 821 ±803 |
| p | - | - | 0.3 | 0.3 | - | 0.7 |
| rs116697954 | CC | 639 | 47.5 ±36.1 | 318 ±105 | 80 | 950 ±703 |
|  | CT | 930 | 48.1 ±35.4 | 313 ±100 | 156 | 918 ±582 |
|  | TT | 361 | 46.2 ±31.9 | 309 ±103 | 50 | 919 ±518 |
| p | - | - | 0.1 | **0.0219** | - | 0.4 |
| rs2037718 | CC | 701 | 47.6 ±33.5 | 314 ±102 | 106 | 953 ±556 |
|  | CG | 970 | 47.3 ±34.8 | 313 ±102 | 160 | 908 ±645 |
|  | GG | 327 | 46.9 ±36.9 | 317 ±97 | 40 | 991 ±666 |
| p | - | - | 0.2 | 0.09 | - | 0.9 |
| rs10216210 | GG | 1,104 | 47.1 ±33.1 | 313 ±102 | 167 | 932 ±605 |
|  | GC | 753 | 48.2 ±36.7 | 316 ±100 | 119 | 958 ±652 |
|  | CC | 141 | 45.2 ±35.6 | 311 ±100 | 20 | 815 ±510 |
| p | - | - | **0.0464** | 0.1 | - | 0.8 |

Metabolic data are shown as unadjusted raw data (means ±SD). Associations between SNP genotypes (additive inheritance model) and insulin secretion were tested by multiple linear regression analyses (standard least squares method) with gender, age, BMI, and OGTT-derived insulin sensitivity as covariates. Nominal associations (p<0.05) are marked by using bold fonts. AIR – acute insulin response; AUC – area under the curve; BMI – body mass index; IS – insulin sensitivity; IVGTT – intravenous glucose tolerance test; OGTT – oral glucose tolerance test; SNP – single nucleotide polymorphism
