# Supplementary material for: Variation in the Phosphoinositide 3-Kinase Gamma Gene Affects Plasma HDL-Cholesterol without Modification of Metabolic or Inflammatory Markers
Source: PLoS One. 2015 Dec 10;10(12):e0144494. doi: 10.1371/journal.pone.0144494 (PMC4675530; doi:10.1371/journal.pone.0144494)
Supplement: S6 Table — (DOCX) [file pone.0144494.s006.docx]

**Table S6. Associations of *PIK3CG* tagging SNPs with plasma glucose concentrations (N_OGTT_=2,066)**

|  | Genotype | N OGTT | Glucose, fasting (mmol/L) | Glucose, 120 min (mmol/L) | Glucose, AUC_0-120 min_ (mmol/L) |
| --- | --- | --- | --- | --- | --- |
| rs4727666 | AA | 1,274 | 5.15 ±0.53 | 6.30 ±1.58 | 14.7 ±3.1 |
|  | AG | 617 | 5.17 ±0.55 | 6.46 ±1.65 | 14.9 ±3.1 |
|  | GG | 94 | 5.15 ±0.56 | 6.20 ±1.60 | 14.7 ±3.2 |
| p | - | - | 0.8 | 0.5 | 0.8 |
| rs3823963 | TT | 680 | 5.17 ±0.54 | 6.32 ±1.57 | 14.8 ±3.0 |
|  | TA | 982 | 5.17 ±0.54 | 6.39 ±1.61 | 14.8 ±3.2 |
|  | AA | 318 | 5.12 ±0.53 | 6.28 ±1.62 | 14.6 ±2.9 |
| p | - | - | 0.2 | 0.9 | 0.2 |
| rs1129293 | CC | 946 | 5.17 ±0.53 | 6.34 ±1.58 | 14.9 ±3.0 |
|  | CT | 854 | 5.16 ±0.55 | 6.34 ±1.60 | 14.7 ±3.1 |
|  | TT | 182 | 5.11 ±0.53 | 6.35 ±1.69 | 14.6 ±3.0 |
| p | - | - | 0.4 | 0.6 | 0.3 |
| rs17401277 | CC | 1,810 | 5.16 ±0.54 | 6.35 ±1.60 | 14.8 ±3.1 |
|  | CT | 196 | 5.13 ±0.54 | 6.25 ±1.66 | 14.5 ±3.3 |
|  | TT | 8 | 5.25 ±0.55 | 6.62 ±0.70 | 15.8 ±2.23 |
| p | - | - | 0.8 | 0.7 | 0.5 |
| rs59813697 | AA | 1,611 | 5.16 ±0.54 | 6.34 ±1.60 | 14.8 ±3.1 |
|  | AC | 364 | 5.17 ±0.54 | 6.38 ±1.62 | 14.8 ±3.1 |
|  | CC | 22 | 5.10 ±0.48 | 6.10 ±0.99 | 14.7 ±2.0 |
| p | - | - | 0.4 | 0.7 | 0.7 |

(continued on next page)

|  | Genotype | N OGTT | Glucose, fasting (mmol/L) | Glucose, 120 min (mmol/L) | Glucose, AUC_0-120 min_ (mmol/L) |
| --- | --- | --- | --- | --- | --- |
| rs4288294 | CC | 748 | 5.14 ±0.55 | 6.34 ±1.64 | 14.7 ±3.1 |
|  | CT | 992 | 5.18 ±0.54 | 6.37 ±1.61 | 14.9 ±3.1 |
|  | TT | 302 | 5.18 ±0.51 | 6.32 ±1.58 | 14.8 ±3.0 |
| p | - | - | 0.06 | 1.0 | 0.1 |
| rs849405 | AA | 1,645 | 5.16 ±0.53 | 6.37 ±1.63 | 14.8 ±3.1 |
|  | AG | 391 | 5.17 ±0.57 | 6.31 ±1.54 | 14.7 ±3.0 |
|  | GG | 30 | 5.14 ±0.60 | 6.10 ±1.58 | 14.1 ±3.1 |
| p | - | - | 0.6 | 0.3 | 0.1 |
| rs116697954 | CC | 663 | 5.13 ±0.54 | 6.30 ±1.57 | 14.6 ±3.1 |
|  | CT | 957 | 5.17 ±0.54 | 6.38 ±1.64 | 14.9 ±3.1 |
|  | TT | 375 | 5.17 ±0.52 | 6.33 ±1.57 | 14.8 ±3.1 |
| p | - | - | **0.0379** | 0.4 | 0.1 |
| rs2037718 | CC | 721 | 5.17 ±0.55 | 6.35 ±1.63 | 14.8 ±3.0 |
|  | CG | 1,005 | 5.17 ±0.54 | 6.40 ±1.61 | 14.9 ±3.2 |
|  | GG | 338 | 5.13 ±0.52 | 6.25 ±1.57 | 14.6 ±3.0 |
| p | - | - | 0.1 | 0.5 | 0.3 |
| rs10216210 | GG | 1,137 | 5.17 ±0.54 | 6.39 ±1.62 | 14.9 ±3.1 |
|  | GC | 782 | 5.16 ±0.54 | 6.32 ±1.58 | 14.7 ±3.1 |
|  | CC | 145 | 5.08 ±0.52 | 6.25 ±1.65 | 14.5 ±3.0 |
| p | - | - | 0.1 | 0.4 | 0.1 |

Metabolic data are shown as unadjusted raw data (means ±SD). Associations between SNP genotypes (additive inheritance model) and plasma glucose concentrations were tested by multiple linear regression analyses (standard least squares method) with gender, age, and BMI as covariates. Nominal associations (p<0.05) are marked by using bold fonts. AUC – area under the curve; BMI – body mass index; OGTT – oral glucose tolerance test; SNP – single nucleotide polymorphism
