# Supplementary material for: Variation in the Phosphoinositide 3-Kinase Gamma Gene Affects Plasma HDL-Cholesterol without Modification of Metabolic or Inflammatory Markers
Source: PLoS One. 2015 Dec 10;10(12):e0144494. doi: 10.1371/journal.pone.0144494 (PMC4675530; doi:10.1371/journal.pone.0144494)
Supplement: S7 Table — (DOCX) [file pone.0144494.s007.docx]

**Table S7. Clinical data of the subgroup with HDL2- and HDL3-cholesterol measurements (N=34)**

| N (women/men) | 21/13 |
| --- | --- |
| Age (y) | 46.0 ±10.4 |
| BMI (kg/m²) | 28.3 ±4.4 |
| Body fat content (%) | 31.1 ±6.4 |
| NGT/IFG/IGT/IFG+IGT | 20/4/4/7 |
| Glucose, fasting (mmol/L) | 5.29 ±0.55 |
| 2-h Glucose (mmol/L) | 7.08 ±1.78 |
| HDL2-cholesterol (mg/dL) | 7.04 ±4.00 |
| HDL3-cholesterol (mg/dL) | 22.6 ±4.1 |
| IHL (% signal) | 6.19 ±5.88 |

Data are given as counts or means ±SD. BMI – body mass index; HDL – high-density lipoprotein; IFG – impaired fasting glycaemia; IGT – impaired glucose tolerance; IHL – intrahepatic lipids; NGT – normal glucose tolerance
